# Complete mitochondrial genome of the great sculpin *Myoxocephalus polyacanthocephalus* (Cottoidei: Cottidae)

**DOI:** 10.1080/23802359.2019.1629845

**Published:** 2019-07-12

**Authors:** Evgeniy S. Balakirev, Alexandra Yu Kravchenko, Pavel A. Saveliev, Alexander A. Semenchenko, Francisco J. Ayala

**Affiliations:** a National Scientific Center of Marine Biology, Far Eastern Branch, Russian Academy of Sciences, Vladivostok, Russia;; b School of Biomedicine, Far Eastern Federal University, Vladivostok, Russia;; c School of Natural Sciences, Far Eastern Federal University, Vladivostok, Russia;; d 2 Locke Court, Irvine, CA, USA

**Keywords:** Mitochondrial genome, phylogenetic relationships, great sculpin *Myoxocephalus polyacanthocephalus*, *Myoxocephalus scorpius*, Cottidae

## Abstract

The complete mitochondrial genome was sequenced in two specimens of the great sculpin *Myoxocephalus polyacanthocephalus* by high-throughput sequencing technology (Ion S5 platform). The genome sequences are 16,651 and 16,652 bp in size, and the gene arrangement, composition, and size are similar to the other sculpin mitochondrial genomes published previously. Overall base composition of the complete mitochondrial DNA is A (26.9%), G (17.0%), C (29.5%), and T (26.6.0%), the percentage of A and T (53.5%) is higher than G and C (46.5%). The difference between the two genomes studied is low, 0.15%. A relatively low level of divergence (3.48%) is detected between *M. polyacanthocephalus* and *M. scorpius*, which however is high enough to consider them as separate biological species.

The great sculpin *Myoxocephalus polyacanthocephalus* (Pallas) is a northwest boreal Pacific benthic species reported from the eastern Sea of Japan to Cape Shpanberga in the Anadyr Gulf, Bering Sea, southern Chuckchi sea, west throughout the Aleutians, and southeast to Puget Sound, Washington at depths of 0–775 m, mostly on the shelf (Neyelov 1979; Allen and Smith 1988; Mecklenburg et al. 2007). The species is widespread and variable in morphology, which make difficult taxonomic identification using morphological criteria (Moreva and Borisenko 2017 and references therein). To increase the power of phylogenetic analysis of this complex fish group, we have sequenced two complete mitochondrial (mt) genomes of *M. polyacanthocephalus* (GenBank accession numbers MK621914 and MK621915) from the northwest of the Malaya Kema river (45.399638°N, 137.213478°E, MPO4-17) and the lake Solenoe (47.17448°N, 138.76151°E, MPO5-17). The fish specimens are stored at the museum of the National Scientific Center of Marine Biology, Vladivostok, Russia (www.museumimb.ru) under accession numbers MIMB 37698 and MIMB 37699.

The genomic DNA was extracted using the KingFisher Flex System and a set of reagents MagMAX DNA Multi-Sample Kit (ThermoFisher Scientific). The complete mt genomes were amplified in five overlapping fragments using the Phusion High-Fidelity DNA Polymerase (ThermoFisher Scientific). Libraries were prepared using Ion Plus Fragment Library Kit and unique adapters (Ion Xpress) with pre-fragmentation on the focused ultrasonicator Covaris M220. Ready libraries were sequenced on the Ion S5 sequencing platform (ThermoFisher Scientific) at the Far Eastern Federal University (Vladivostok, Russia). The complete mt genomes obtained were initially annotated using the MitoFish Web Server (Iwasaki et al. 2013) and further manually adjusted with MEGA 7 (Kumar et al. 2016) by comparisons with mt genomes of other sculpin fishes.

The *M. polyacanthocephalus* mt genomes (120× coverage) are 16,651 and 16,652 bp in size; the gene arrangement, composition, and size are very similar to the sculpin fish genomes published previously. Overall base composition of the complete mitochondrial DNA is A (26.9%), G (17.0%), C (29.5%), and T (26.6.0%), which indicated an AT bias (Shadel and Clayton 1997). There are 26 single nucleotide and one 1-bp length differences between the two haplotypes of MPO4-17 and MPO5-17; total sequence divergence (*D*_xy_) is 0.0015 ± 0.0003. Comparison of the two mt genomes now obtained with other complete mt genomes available in GenBank for the genera *Myoxocephalus*, *Enophrys, Icelus*, *Trachidermus*, *Mesocottus*, and *Clinocottus* reveals a close affinity of *M. polyacanthocephalus* to a congeneric species *M. scorpius* (Li et al. 2019) (Figure 1). The difference (*D*_xy_) between *M. polyacanthocephalus* and *M. scorpius* is 0.0348 ± 0.0011, which is high enough to consider them as separate biological species.

**Figure 1.**
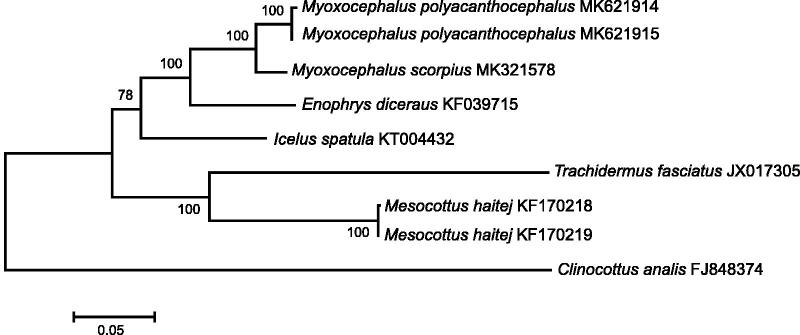
Maximum likelihood tree for the great sculpin *Myoxocephalus polyacanthocephalus* and GenBank representatives of the family Cottidae. The tree is constructed using whole mitochondrial genome sequences. The tree is based on the Hasegawa-Kishino-Yano + gamma + invariant sites (HKY + G + I) model of nucleotide substitution. The numbers at the nodes are bootstrap percent probability values based on 1000 replications.
